# Evaluation of MCQs from MOOCs for common item writing flaws

**DOI:** 10.1186/s13104-018-3959-4

**Published:** 2018-12-03

**Authors:** Eamon Costello, Jane C. Holland, Colette Kirwan

**Affiliations:** 1 Dublin City University, Glasnevin, Dublin 9, Ireland; 2 RCSI Dept of Anatomy, 123 St Stephens Green, Dublin 2, Ireland

**Keywords:** MOOCs, MCQs, Quality, Item writing, Tests, Education, Pedagogy

## Abstract

**Objective:**

There is a dearth of research into the quality of assessments based on Multiple Choice Question (MCQ) items in Massive Open Online Courses (MOOCs). This dataset was generated to determine whether MCQ item writing flaws existed in a selection of MOOC assessments, and to evaluate their prevalence if so. Hence, researchers reviewed MCQs from a sample of MOOCs, using an evaluation protocol derived from the medical health education literature, which has an extensive evidence-base with regard to writing quality MCQ items.

**Data description:**

This dataset was collated from MCQ items in 18 MOOCs in the areas of medical health education, life sciences and computer science. Two researchers critically reviewed 204 questions using an evidence-based evaluation protocol. In the data presented, 50% of the MCQs (112) have one or more item writing flaw, while 28% of MCQs (57) contain two or more flaws. Thus, a majority of the MCQs in the dataset violate item-writing guidelines, which mirrors findings of previous research that examined rates of flaws in MCQs in traditional formal educational contexts.

## Objective

Despite increasing debate about the potential for Massive Open Online Courses (MOOCs) to contribute to formal, accredited qualifications, there is an absence of research into the quality of their assessments, including those based on Multiple Choice Question (MCQ) items. This provided the motivation to undertake an exploratory study of a selection of MOOCs to determine the existence and prevalence of item writing flaws in their MCQs. The full study and its findings are reported elsewhere [1, 2], but not the associated dataset provided here. We collected data from a sample of MOOCs (18) in the areas of medical health education, life sciences and computer science, and two researchers critically reviewed these 204 MCQ items using an evidence-based evaluation protocol derived from the medical health education literature [3]. Item writing flaws have been shown to compromise the reliability and validity of assessments and their outcomes, with inconsistent and unpredictable effects [4–6]. For instance, one study found that 33–46% of questions in a series of science examinations were flawed, potentially incorrectly failing 10–15% of examinees who should have passed [4]. Another study found the converse, that some students who passed nursing examinations incorporating flawed items should arguably have failed [5]. Hence the data described here was generated with the objective of ascertaining whether such flaws pertain in MOOCs and to what degree.

## Data description

The dataset incorporates assessments of human evaluators regarding MCQ quality in a selection of MOOCs. A set of 204 MCQs were collected by manually recording the questions, the options (or distractors) posed as potential answers and the actual correct answer from 18 MOOCs, and inputting these data into a spreadsheet. Two evaluators then independently reviewed the 204 MCQs, using an evaluation protocol adapted from Tarrent et al. [3] and which we include under workbook “Evaluation Instrument”. We did this through a Google Form. A unique id was assigned to each MCQ of the format q*i*–*j*, where *i* is the quiz to which the MCQ belongs and *j* is the number of the question in that quiz. We then pre-populated the Google form with a drop down menu of these identifiers. Each evaluator then selected the identifier of the question they were working on, and proceeded to fill in the form with their evaluations, which simplified and synchronised the workflow.

This led to the generation of the spreadsheet table which records evaluations for each of the 204 MCQs by each evaluator, considering 15 specific item flaws. For example, in the workbook “Evaluator 1 responses” cell A21 has the value “q11-7” which indicates it is the seventh question from quiz 11 in the data. In cell G21 the value “no” is recorded which tells us that evaluator 1 was of the opinion that this MCQ question did not contain plausible distractors. While the determination of what constitutes a plausible or implausible distractor is to some degree subjective (although may be supported by quantifiable data from statistical item analyses if available), the evaluators making this judgement were content experts. Implausible distractors can make it easy for student to guess the correct answer, as the correct option stands out as being the only obvious choice [7]. In the next workbook “Evaluator 2 responses” we can see that evaluator 2 recorded the same evaluation. The evaluators then compared their results, discussed any evaluations where their conclusions differed, and then agreed a final evaluation by consensus. These results are recorded in the “Combined Evaluations” worksheet.

Additional item writing flaws exist that can be identified without a human evaluator. These are: the number of possible correct options (1 is optimal); whether the correct option is the longest, as the longest is often the correct answer (these were calculated programmatically by counting the number of characters in each option); the number of options (3 or 4 are considered optimal [8]); whether “all of the above” or “none of the above” are options or not, as these violate best practice in item writing; and lastly, the position of the correct option (as research indicates option 3, or C, to most often be correct). These data points for each MCQ are recorded in the “Quantitative items” workbook. The workbook “Table 1 flaw prevalence” gives summary descriptive statistics of the raw results presented in the other workbooks. The final workbook, “MOOC platform and institutions”, lists the platform and the institution of each MOOC.

**Table 1:** Overview of data files/data sets

Label	Name of data file/data set	File types (file extension)	Data repository and identifier (DOI or accession number)
Data file 1	Dataset MOOCs MCQs study v2.xlsx—3.0 MB	Excel file	Zenodo 10.5281/zenodo.1487858

## Limitations

Our sample of MOOCs and MCQs contained therein is a relatively small one. We had specific inclusion criteria and there were some practical limitations to our data collection. We examined only MOOCs delivered through the English language. We confined ourselves to topics in which the researchers’ had expertise i.e. focused in health sciences and computer science. We enrolled in MOOCs to take the quizzes which was a labour-intensive data gathering approach. Consequently, we sampled those courses that happened to be enrolling during the data collection window according to a convenience sample i.e. not a true random sample. However, we believe these data from this exploratory research to be potentially useful to others, given the dearth of research in this area, the growing importance of MOOC assessment (including MOOCs for formal credit) and the high prevalence of errors we found.

Most of our evaluations are qualitative in nature. To overcome this limitation two evaluators worked independently to perform their evaluations, and then met to compare results. Inter-rater reliability was calculated (Cohen’s Kappa score of 0.92) which indicated high agreement at this stage. Then the evaluators reviewed the items that were not in agreement and created revised combined scores.

We do not include the data on the questions in the MOOC quizzes themselves as they are copyright of the MOOC platform providers. However, we are happy to share further aspects of these data upon request to interested researchers.

## Abbreviations

MOOC: Massive Open Online Course
MCQ: Multiple Choice Question
